# Unsatisfactory colposcopy: clinical decision-making in conditions of uncertainty

**DOI:** 10.1186/s12911-017-0516-3

**Published:** 2017-08-22

**Authors:** Kristyn M. Manley, Rebecca A. Simms, Sarah Platt, Amit Patel, Rachna Bahl

**Affiliations:** 1 Department of Women’s Health, University Hospitals Bristol NHS Foundation Trust, St. Michael’s Hospital, Level D, St. Michaels Hospital, Bristol, BS2 8EG UK; 2 University of Bristol, Bristol, UK

**Keywords:** Unsatisfactory colposcopy, Attitudes, Decision-making, Clinical uncertainty

## Abstract

**Background:**

Unsatisfactory colposcopy, where the cells of interest are not visible in women with a positive cervical screening test, is a common area of clinical uncertainty due to the lack of clear evidence and guidance. Colposcopists’ opinions and experiences are likely to have a significant influence on service provision and the development of national policy. The aim of this study was to analyse decision-making when applied to women with unsatisfactory colposcopy.

**Methods:**

A multi-centre qualitative study utilizing a series of focus groups in an English healthcare region. Sampling aimed to ensure heterogeneity of experience and healthcare provider demographics. A topic guide covered a range of clinical and cytological variables and was compiled by the researchers and three expert Colposcopists. Using an iterative approach, thematic analysis was selected as the most appropriate method to identify factors affecting decision-making.

**Results:**

Twenty-three Colposcopists from four units participated. The decision to treat was easier in women with high-grade cytology and high risk women with low-grade cytology such as heavy smokers, poor attenders, older women, those who had completed their families and women opting for treatment. Where decision-making was more complex, intuition and a multi-disciplinary approach were used to guide management. Areas of dissonance, which are affected by paucity of evidence and emotive factors, included cytological collection device, clinical setting and length of conservative follow-up and depth of excision in women at high risk of treatment-related morbidity.

**Conclusions:**

Anxiety of missing a cancer deters long-term cytological follow-up, resulting in heterogeneity of care and higher than anticipated excisional treatments in women with low-grade screening and unsatisfactory colposcopy. In areas of clinical uncertainty when decisions are dominated by affect, clinical guidance can reduce the difficulty and anxiety of decision-making.

## Background

Persistent infection with Human Papillomavirus (HPV) causes 99% of cervical cancers [1]. In the UK, primary HPV screening was instituted in six ‘sentinel sites’ in 2011 (including Bristol) and the high negative predictive value of this test (>99%) [2] has led the NHS Cervical screening programme to recommend replacement of the current HPV triage of low-grade cytology with HPV primary screening. The screening sample will initially be tested for one of the 30 high risk HPV subtypes and if positive, a woman’s risk of pre-cancerous change will be triaged into low-grade or high-grade by a cytology test. This result helps to stratify who may require treatment; with a HPV positive, high-grade cytology result there is an 82% chance that the excised tissue will contain high grade pre-cancerous cells and a 2.6% chance of cancer. With a HPV positive, low-grade cytology result, this risk is 15.9% and 0.1% respectively [3].

However, a positive screening result is not a definitive determinant of outcome. The positive predictive value (PPV) of detecting CIN2+ in women who have high risk HPV and low-grade cytology is only 16% [2]. 80% of immunocompetent women will clear a HPV infection [4] and the low PPV indicates that HPV testing currently fails to differentiate between these women and the 20% who will have persistent infection.

Therefore, women with a positive cervical screening test are referred to the colposcopy clinic. The purpose of this assessment is to visualise the area infected by HPV and determine who requires treatment and who can be managed with conservative cytological follow-up. Management difficulties arise when the cells of interest are ‘tucked inside’ the cervix and are not visible for assessment. This is known as unsatisfactory colposcopy or a transformation zone (TZ) type 3, the incidence of which is approximately 20% [5], potentially accounting for 34,555 of the 172,776 women reviewed in Colposcopy each year [3]. The identification of a TZ type 3 and the inability to provide histological selection for treatment may deter cytological follow-up and lead to higher rates of excisional treatments in women with low-grade screening results [6] to prevent missing a ‘hidden’ cancer.

The quandary for Colposcopists is the treatment related morbidity as a deeper excision (15-25 mm) is recommended in this cohort [7]. This treatment can increase the risk of preterm birth [8] and cervical stenosis which in turn can lead to infertility and difficulty with future cytological assessments [9, 10]. Furthermore, there is an 8.6 fold increased risk that the excised tissue will be normal when compared to excisions where the cells of interest are visible [6]. It seems clear that a reduction in unnecessary treatments is needed to improve patient outcomes.

Currently, guidance for cytological follow-up versus excisional treatment is hospital specific [5, 7, 11] in this cohort and this may lead to disparities in care. Attendance rates for colposcopy and loss of patients to follow-up has been shown to be affected by service inefficiencies [12], anxiety [13, 14] and poor accessibility to targeted information [15, 16]. With non-attendance rates for colposcopy in the UK documented at 24.4%, of which 46.1% are follow-up appointments [17], areas of heterogeneity in service provision need to be improved.

Clinical decision-making is a complex process and the inconsistent nature of intuitive management has led to the development of evidenced based practice [18] which aims to minimise morbidity and maximise optimal outcomes. However, when a paucity of evidence exists, decision-making under conditions of uncertainty can be influenced by patient choice or demographics and health care provider attitudes, experience, age, gender or culture [19, 20]. Colposcopists play an important role in leading research and policy change in cervical screening programmes and there is currently no literature to suggest how their opinions and experiences shape the management or counselling of these patients. The aim of this study was to identify factors that affect Colposcopist’s decision-making, specifically recommendations for excisional treatments over cytological follow-up, and to interpret these findings in line with decision-making theory.

## Method

This was a qualitative study, utilising a series of focus groups. Colposcopists working in NHS trusts within the South West of England Region were purposively sampled to achieve maximum variation [21]. Participants were included if they were active accredited members of the British Society for Colposcopy and Cervical Pathology (BSCCP); membership is a pre-requisite to practice as a Colposcopist as the BSCCP standardises training and audits quality of service provision. In two waves of recruitment, lead Colposcopists from each trust (who are responsible for quality assurance) were contacted by email. They forwarded the study information to all Colposcopists within the unit to request participation. Prior to taking part in the focus groups, Colposcopists’ provided written consent to participate, to be audio-recorded and for anonymised quotes to be utilised in publications.

Sampling aimed to ensure heterogeneity, such that a range of demographics and opinions were included to improve the generalisability of the findings. Data regarding years of experience in colposcopy and job title were collected. Different health care professionals such as nurses, oncologists, family doctors and general gynaecologists can accredit as Colposcopists; their background experience may shape their attitudes and opinions and affect the decisions they make. Protocols and educational experience may vary in different units and therefore demographic location of the Colposcopists’ training centre was also collected. To maintain anonymity, age and gender were not collected. Data saturation [22], such that no new opinions or attitudes were identified, was achieved with a total of twenty-three Colposcopists from four centres. At this point, recruitment ceased.

A topic guide (Table 1) was designed by qualitative and clinical researchers in collaboration with three experts in the field (who did not participate in the focus groups). An expert was defined as a Colposcopist who was respected and nominated by their peers for their expertise in colposcopy, as these practitioners manage complex as well as routine cases. The topic guide consisted of open questions; these aimed to focus the discussion and allow an exploration of the decisions that are made when reviewing women with unsatisfactory colposcopy and a range of clinical and cytological variables. These questions focused on length, clinical setting and technique of follow-up, the depth of excision undertaken in relation to patient and clinical characteristics and how Colposcopist’s practice is influenced by the current literature and guidance. These semi-structured focus groups enabled the researcher to cover the core set of questions and allow a flexible and dynamic discussion that could be led and expanded upon by the participants. To understand the Colposcopists’ decision-making process, participants were asked to identify the criteria they used in management decisions by asking ‘why’ and ‘how’.

**Table 1:** Focus Group Topic Guide

Unsatisfactory Colposcopy Topic Guide	
Conservative management - Why - Effect of age - Effect of parity - Effect of HR HPV - Length of follow up - Place of follow up - Technique of follow up (what)	Depth of LLETZ - How deep? - Why? - Who? - Alternative methods of treatment
Who to treat? - How - Where - Why - Effect of age - Effect of parity - Effect of HR HPV	Quality monitoring - Reporting methods - Interpreting the reports - HPV education of colposcopists
Oestrogen - Who? - How? - Alternative uses - Other methods of everting the TZ?	Issues regarding colposcopy management - National guidance - Patient focused.

A researcher (KM, who is an accredited Colposcopist and trained in qualitative research methodology) conducted the focus groups. Due to the amount of technical content, the challenge of conducting these discussions was reduced by KM being a Colposcopist. A facilitator (SP or RB) were present to aid transcription by recording the speaker order, noting non-verbal communication and assisting in further exploration of raised points. The focus groups were composed of staff from each individual trust (block sampling), conducted in private rooms at the participants’ hospital and lasted 40–50 mins. Refreshments were provided to facilitate participation during Colposcopists’ lunch times. Field notes were written immediately after the focus group to aid in the interpretation of the transcripts and contextualise the discussion. Interviews were transcribed (by KM) with the aid of the speaker list. All participants were anonymised and the transcripts sent to respondents and the facilitator (SP/RB) for validation. One participants’ statement was refined to expand upon how their anxiety of missing a cancer affected their decision-making, but this did not lead to the researchers disregarding their interpretation of the data. The qualitative software package NVivo 10 was used to aid analysis.

Thematic analysis (TA) was selected as the most appropriate method for this study; it is one approach that can be used to identify, analyse and organise patterns of opinions within a data set. We chose this method as the experiences of participants can be analysed without evaluating how they experience reality (such as with IPA or phenomenology). It also provides the flexibility to allow participants to expand upon their worries and interests without deviating from the decision-making process (which was the aim of this study). TA was chosen in preference to grounded theory as data was collected through the use of focus groups and the focus was not on social processes [22, 23]. Data was inductively coded, using the six stage TA process described by Braun and Clarke [24]; as outlined below.

In an iterative process, analysis was conducted after each interview so that future focus group questions were informed by prior analysis [22]. After familiarisation with the data and discussion between the researchers, a coding list was developed and the first transcript individually coded by three researchers (KM, RS, RB). RB and RS are postdoctoral qualitative researchers who are affiliated with the University of Bristol and have no clinical involvement in the Colposcopy units that were approached. The data informed the coding, rather than using a rigid pre-designed coding structure or framework of behavioural determinants, to reduce the chance of the researcher’s pre-conceived ideas affecting the themes that were identified in the data set. The coding framework was then applied to future transcripts and revised once more as further transcripts were analysed. To achieve a rigorous analysis, consistency of interpretation was assessed: Two research members (KM and RS) independently coded the last three transcripts, results were compared, divergences discussed and disagreements settled by a third researcher (RB). Field notes were compared with the transcripts to define tone and potential meaning of the words transcribed.

On completion of the coding, all researchers met to discuss and refine key themes that were described in the data. Themes were then defined following in-depth consideration of potential alternative interpretations through the use of mind maps and iterative lists. In a semantic approach, illustrative quotes, descriptive accounts and tables of the themes were then developed from the data. A framework of decision-making was developed after a literature search and brainstorming. Themes that were identified within the data were then mapped to the relevant theoretical constructs within this framework.

## Results

Twenty-three of a potential twenty-eight Colposcopists from four units participated in four focus groups. The Colposcopists who declined gave conflict with their clinical workload as the reason for non-participation. The participants represented a range of years of experience, geographical training backgrounds and specialty. There were five nurse practitioners, four gynaecological oncologists, three lead Colposcopists, seven gynaecology consultants, two pathologists and two gynaecology registrars. Years of experience in colposcopy ranged from 1 to 34 with the mean number of years 11.2. Fourteen participants trained in the South West region of the UK, three in London, two in the West Midlands, two in the Northern region, one in the North East and one in the Eastern region.

Two of the units were included in the sentinel sites study and had eight years of experience in managing women referred with HPV triage of low-grade cytology and three years of experience with primary HPV testing prior to this study’s data collection. The remaining two units had two years’ experience of HPV triage of low-grade cytology following the national roll-out in 2013. Four key themes which affected decision-making were identified;

### Theme 1: anxiety of missing cancer

#### Lack of confirmatory histology

In our study, irrespective of a high-grade or low-grade result, if the cells of interest were not visible and a diagnostic biopsy was not possible, most participants were deterred from advocating long-term cytological follow-up.

**01:** ‘They’ve come to colposcopy and it’s been pointless because you’re not getting the information you want from that examination. Yes, there is a good chance it could clear up but I just feel a bit nervous about leaving them because there could be high grade there that you can’t see...It’s knowing whether it’s there or not, that’s the nightmare.’

This suggests an affective component to decision-making; fear of missing high-grade disease induces a pessimistic outlook of future events if women are not offered an excisional treatment. It appears that this is of higher importance than concerns relating to treatment morbidity, even in women with a low-grade cytology result.

#### Impact of a high-grade cytology result

In these women the majority of participants strongly advised, with patient consent, an excisional treatment irrespective of age or family status. Affect and cognition had a major influence on decision-making; anxiety of missing a cancer if treatment was not undertaken was driven by the plethora of evidence which shows there is a significant risk of the excised tissue containing high-grade pre-cancerous cells. When making decisions that could result in dangerous outcomes and negative emotions, participants made safe choices.

**02:** ‘If you think of these women having a cancer, there’s a cancer that we just can’t see, it’s tiny, it’s inside the canal. If you repeat the cytology and you are giving it at least three months, then we don’t know if those three months could make a 1a1 (Cancer grading) into a 1a2 or even a 1b1 - who knows that? So that’s my worry, I look at what’s the worst possible scenario here and with high grade cytology it’s more than likely that there will be high grade disease inside.’

### Theme 2: the screening test result

#### A HPV positive status increases the risk of treatment

Although Colposcopists did not reflect on the global impact of HPV testing within colposcopy, they did discuss how a high risk HPV result in conjunction with low-grade cytology would affect their decision-making. Sixteen Colposcopists suggested that a high risk HPV result increased the chances of a woman having underlying high-grade disease. This belief appeared to reduce the uncertainty in their decision-making as all participants agreed that this perceived increase in risk, with the potential of missing a cancer, was leading to higher rates of excisional treatments in this cohort.

**22:** ‘I think, in the younger ones, how long do you wait to see if the HPV is going to resolve? And I think that now we are using HPV testing that has upped the ante. So you know that they’ve still got active HPV and the longer that stays the more likely they are to have an abnormality. So I do talk to them about having a LLETZ (excisional treatment).’

**07:** ‘I think, you know, the sensitivity has gone up and you do see high-grade histology with low-grade smears...and I would probably, in the older women, I would be much more pushed to do just a small LLETZ.’

Colposcopists appear to be highly risk adverse and choose, what they view, as the least dangerous outcome i.e. treatment morbidity over the chance (even if small) of missing a cancer. Being ‘pushed’ into advocating a treatment suggests they would have preferred to manage women with low-grade screening results more conservatively but worried about the potential risks of doing so. Pathologists and lead Colposcopists (who are responsible for quality assurance) suggested that Colposcopists’ behaviour was shaped by their experience of reviewing women with a positive HPV result. Rather than recognising the benefits of an improved screening test, they suggested other Colposcopists felt women were at increased risk of high-grade disease and this conferred the belief that conservative follow-up was a riskier choice.

**14:** ‘There is a shift in expectations and opinion rather than pathology.’

**21:** ‘What you’re doing is weeding out the women with borderline or low-grade smears who have no pathology because they are HPV negative...a patient who has a mild smear with HPV positive is at no greater risk than they were 10 years ago.’

### Theme 3: patient characteristics

#### Stratifying risk factors for high-grade disease

When patients present with individual characteristics known to increase the risk of cancer such as smoking, poor attendance, older age and / or increased parity, behavioural decision-making is influenced by analytical thinking. Rational judgement denoted that in women with low-grade screening, the decision to treat was easier if women were stratified as high-risk - the possibility of developing a cancer outweighed the risk of treatment-related morbidity.

**14:** ‘If she’s a heavy smoker and she’s clearly never going to give up, then that predisposes me towards treatment...’

**04:** ‘...I think you’re going to be more likely to do a small LLETZ (excisional treatment) on someone you’re going to be concerned about their attendance. It’s an individual thing.’

**01:** ‘If she had had her family or was an older lady, I would do a loop (excisional treatment) for diagnosis.’

#### A multi-disciplinary approach

Young women who have not started their families and present with low-grade cytology and risk-adverse behaviour are at low-risk of high-grade dysplasia but high risk for treatment related morbidity. A multi-disciplinary approach was advocated by fourteen colposcopists in three focus groups for these women.

**13:** ‘A young nulliparous woman I would bring to the MDT to get a consensus that it was the right thing to do a LLETZ (excisional treatment), to be honest, in case they have problems in the future. If it was an MDT decision I’d feel happier.’

This suggests participants are concerned that their affect is influencing their rational thinking and by sharing decision-making with an expert group they assuage this emotional response. The participants who advocated the MDT suggested this choice was influenced by the introduction of HPV screening. They indicated this had resulted in women being referred to colposcopy earlier, reducing the time needed to clear the infection and subsequently increasing the chance of over-treatment. Cognition (their knowledge of the natural history of HPV infection) and intuition (prior experience of reviewing women pre- and post-HPV screening) appears to affect behaviour.

***16:*** *‘We’re treating them sooner because they are coming to us sooner.’*

***14:*** *‘But that’s a potential disadvantage isn’t it....?’*

***16:*** *‘Not if there’s high grade in it.’*

***14:*** *‘But, we might be over-treating the women who potentially might get better.’*

***17:*** *‘CIN2* (pre-invasive disease) *in a young girl has a 40% chance of regression, doesn’t it.’*

#### Patient choice

This was discussed as a major factor in decision-making in all of the focus groups. Colposcopists acceded to patient treatment wishes, even in women at high risk of treatment morbidity and low risk of disease, if the woman was informed of and understood the potential risks of their chosen management option. This suggests that cognitive factors influencing Colposcopist’s decision-making can be superseded by the patient’s affect and cognition.

***03:*** *‘You know, it’s a discussion with the patient explaining the pros and cons”.*

***17:*** *“I had a woman the other day, actually, who wanted a LLETZ* (excisional treatment)*. Her mum died of cervical cancer, she actually had a low grade smear*, *but she said ‘just cut it away’. I was like ‘but you’re 25, let’s just do a couple of smears’ and she said ‘just cut it away’. So I spoke to the consultant on call and they said ‘do what the patient wishes’”.*

None of the participants mentioned scenarios in which women had declined treatment and the subsequent impact of this choice.

### Theme 4: paucity of guidance engenders reliance on clinical experience

#### Clinical setting for cytological follow-up

In women with low-grade screening results who were deemed suitable for cytological follow-up, there was a lack of consensus regarding where to review them. Eight participants (including four of the five nurse Colposcopists) advocated GP (family doctor) follow-up.

**09:** ‘If they’ve got abnormalities already on the current smear, there’s no good reason why you should have to bring them back to colposcopy. If it was a low grade smear I would probably send her back to her GP because we’ve got direct referral to colposcopy and we all use the same lab.’

This suggests trust in the reliability of the laboratory and a belief that outcomes will be the same in the community and the hospital as smear technique is standardised. Conversely, fifteen colposcopists, who were all doctors, differed in this opinion:

**12:** ‘I hear what you’re saying about having a good reliable lab but patients aren’t often very reliable and so I’d like to know that she’s been followed up and make efforts to do so.’

**07:** ‘We haven’t made the diagnosis yet, she still sort of belongs to us. We can’t discharge her to the community without working out whether there is something to get concerned about or not.’

The doctors suggested they had a responsibility to ensure a decision / diagnosis was made and a cancer not missed by personally reviewing these women. Doctors tend to review more complex cases and the adverse outcomes of missed diagnosis. This may have influenced their management choice. It appears that emotion can be more influential that cognitive elements when the risk of cancer is factored into decision-making. The role of emotion and responsibility was strengthened by the paucity of guidance surrounding the optimal technique of taking a smear in women with unsatisfactory colposcopy.

**17:** ‘They don’t do endocervical smears (in the community) so how can you specify that? You see I would do a cytobrush and broom. With a type 3 transformation zone you’re more likely to get a better specimen with a brush and broom, aren’t you?’

**14:** ‘I agree with you, but has anyone proven that.’

**17:** ‘No, not that I know of.’

It appears that in conditions of clinical uncertainty, intuitive decision-making - affect, perception, rational judgement and prior experience – aids Colposcopists in assessing risk.

#### Length of cytological follow-up

For those women whom participants recommended cytological follow-up rather than excision, there was a discrepancy in the number of months advocated before repeating the smear. Thirteen colposcopists suggested six months and if at this time, any grade of cytological abnormality was reported and the examination was still unsatisfactory, they would recommend an excision.

**16:** ‘I would prefer to see them in 6 months. They’ve come to colposcopy and it’s been pointless because you’re not getting the information you want from that examination. Yes, there is a good chance it could clear up but I just feel a bit nervous about leaving them because there could be high grade there that you can’t see.’

**03:** “…I do think it’s kind of two strikes and you’re out, the referral cytology and a 6 month follow up, because they clearly still have got continuing HPV”.

This suggests an affective component to decision-making; anxiety about missing high-grade disease, compounded by the perceived risk that persistent HPV confers, deterred long-term follow-up even in women with low-grade screening. Conversely, six colposcopists discussed individualising care based on patient risk factors. If women were young and/or nulliparous with low risk factors, participants recommended a 12 month cytological follow-up.

***09:*** *‘The 12 month repeat allows the immune system to battle HPV, as studies showed there is a greater clearance at 12 months rather than 6 months.’*

***14:*** *‘The debate we’re having here is whether 6 months or 12 months is better and the issue or question is whether this lady might have a high grade dysplasia underlying. The likelihood of that becoming a malignancy in the 6-12month phase is (pause) in the order of a fraction of a percent.’*

This suggests a combination of cognitive and intuitive decision-making based on prior experience, perception of risk and knowledge.

### Repetition of the referral cytology

Two of the Colposcopists who could not attend the focus groups provided the researchers with a scenario they considered an area of clinical uncertainty; due to the topographic position of the TZ, would Colposcopists repeat the referral cytology at the first colposcopy appointment? The majority of the Colposcopists adhered to national guidance and did not repeat the smear. However, some participants suggested they had concerns that the referral cytology collection device may not have adequately sampled the TZ due to its endocervical position.

**01:** “I think if we speak to any cytologist they’ll always say you should not repeat the smear within 8 weeks because you’ve already sampled it and you’ve already taken off the epithelium and then you really need to wait for it to re-grow or you’re going to get a false positive / false negative and you’re going to be back to square one”.

**16:** “I would wait three months. I know not everybody does”.

**17:** “If it was a poor sample with a TZ3 then I would re-smear”.

### Oestrogen use is based on prior experience

The use of oestrogen has been discussed in the literature as a potential pharmacological method which can convert unsatisfactory colposcopy to satisfactory. Colposcopist’s discussed their recommendations for its use which appeared to be linked to prior experience.

***12:*** *‘The thing with oestrogen is you’re never too sure about the compliance prior to it and whether that makes a difference.’*

***01:*** *‘If she has an atrophic cervix I would ask her, definitely, to have two weeks of oestrogen before her next cytology...not so much because you’re going to pull out the transformation zone but, it can help the interpretation of the cytology. Also for her comfort...’*

For this intervention, it appears that intuition plays a greater role than cognition. Despite the evidence suggesting the potential benefits of use the majority of participants felt, in practice, it did little to improve the examination findings. The majority of gynaecological oncologists did not advocate use but eleven participants, including all of the nurse colposcopists, prescribed oestrogen to improve the smear quality and reduce discomfort during the examination. Gynaecological oncologists manage women with oestrogen driven cancers and this may have affected their decision-making, particularly as they reported no real improvement in examination adequacy and therefore the harm of oestrogen use may have outweighed the benefit in their minds. Three of the four units used topical preparations and the reasons identified were the side effect profile and poorer efficacy of systemic hormone replacement therapy. As with seen in theme one, negative emotions led participants to make, what they considered to be, the safer management options.

***06:*** *‘Well I guess topical oestrogens are less harmful than actually giving HRT...and it works*.’

### Depth of excisional treatment (LLETZ)

Decision-making in this area was driven by prior experience, perceived individual risk and affect.

**14:** ‘Greater than 7 less than 10mm to reduce the risk of cervical dysfunction in pregnancy.’

**07:** ‘There’s a chance there’s absolutely nothing wrong with her cervix and you’re chucking out a big bit of tissue and if you do really have something wrong with the cervix there’s the option of doing a second LLETZ if you’re really concerned.’

**02:** ‘I think the biggest problem is that because you can’t see the TZ (cells of interest) you don’t know how far to go...If you do a deeper loop and it is negative that is much easier to criticise than if you do a smaller loop and then if it is positive, you do another one because that is much more targeted.’

As seen in theme one and with systemic oestrogen use, Colposcopists make safe choices. Women at high-risk of treatment related morbidity engender negative emotions which prompted participants to make autonomous choices and deviate from UK national recommendations for optimal depth (15-25 mm) [7]. In older woman, who are at reduced risk of treatment morbidity and increased risk of high grade disease, Colposcopists adhered to national guidance. Cognition and rational judgement had a greater impact than affect in this patient demographic.

**13:** ‘In an older woman I probably would go a bit deeper because they’re more likely to have an adenocarcinoma than squames.’

**04:** ‘The older women, 15mm, what you want to avoid, if possible, is the inconvenience of bringing them back for a repeat LLETZ and risking non-attendance.’

Conditions of clinical uncertainty can cause anxiety in both health care providers and patients. The use of rational judgements and Colposcopist’s experience appears to aid in decision-making but affect appears to play a strong (and sometimes more dominant) role when evaluating risk. The following quote most accurately reflected the overall findings of this study:

**03**: ‘I think it’s interesting. I think what we’re all talking about is individualisation of care . . . All you’re trying to do is be safe to gain or achieve the information that you need and it does need to be individualised. And I think in our day to day practice that’s what we all spend our lives doing.’

## Discussion

Excisional treatments have helped reduce the overall mortality rate from cervical cancer by 60% in the UK [17] but the benefits of treatment have to be tempered with the associated morbidity. Women with unsatisfactory colposcopy and low-grade cytology have higher than desired treatment rates [6] and it is therefore important to explore factors which may influence decision-making in this cohort. This paper addresses an important issue – the ways in which medical practitioners, in this case Colposcopists, make decisions under conditions of uncertainty. A qualitative approach sheds a useful light on the process of decision-making and to the best of our knowledge this is the first study which addresses this issue. Where rational judgement, cognition and affect could be applied, areas of consensus were identified; A multidisciplinary team decision, patient preference, a high-risk screening result or a low-risk result in combination with patient risk factors such as poor compliance, smoking, high parity or older age resulted in recommendations for excisional treatments. In areas of clinical uncertainty Colposcopist’s experience, knowledge, rational judgement, perception and affect influenced decision-making. When faced with an inability to provide colposcopic assessment or diagnostic histology the psychological stress of missing a cancer, even in women with low-grade screening, deterred prolonged or community based cytological follow-up. A paucity of guidance and patient anxiety further compounded decision-making and led to heterogeneity in care.

Decision-making is a complex process which incorporates knowledge, risk assessment, analytical skills, prior experience and affect [25]. Decision-making can be challenging in areas of clinical uncertainty where guidance is sparse [26, 27], when an adverse outcome such as a cancer may occur as a result of the decision [28] or if a large number of variables need to be contemplated when making a decision [26].

These themes were illustrated in our study when participants, particularly Gynaecological Oncologists, suggested that the possibility of removing high grade disease outweighed the risk of treatment-related morbidity in women with significant risk factors. In women with low-grade cervical screening, the TOMBOLA study [29] advocates a policy of surveillance rather than immediate treatment to allow regression of pre-invasive disease. This policy however relies on colposcopic visualisation and histological confirmation of the lesion, which cannot be undertaken in women with unsatisfactory colposcopy. Whilst conscious of the risk of over-treatment, particularly in younger women, participants were more concerned about missing a developing cancer. This finding is supported by studies which have shown that in areas of uncertainty, decisions are made faster and more easily by relying on emotion [30]. Furthermore, when an emotive thought, such as fear of missing a cancer, induces anxiety, this can lead individuals to place more weight on the negative outcomes than the positive [31, 32]. Once distracted by a negative stimulus it is then difficult to divert attention from these negative thoughts [33]. Anxiety has been associated with increased amygdala and reduced pre-frontal activity [34] which suggests that in areas of uncertainty affective components of decision-making may take precedence over rational cognitive elements [35, 36].

In our study, uncertainty of decision-making in women with low-grade cytology was reduced by the perceived increase in risk that a persistent HPV result conferred. However, recent evidence has shown that the proportion of women with a low-grade screening result and subsequent grades of pre-cancerous disease (CIN 1, 2 or 3) is no different following the introduction of HPV testing [3]. What has fallen is the number of women referred to colposcopy with inadequate or borderline results and those with normal colposcopy [3]. This could be falsely viewed as an increase in individual risk, leading to a more aggressive management approach when histological selection for treatment is not possible. Most people are naturally risk adverse and look to avoid poor outcomes by selecting the least risky option [37]. Uncertainty of outcome (inability to visualise the transformation zone) heightens anxiety and compounds this risk aversion. When it is not clear whether the alternative decision may result in further risk or benefit, willingness to take a risk, in this case prolonged cytological follow-up, is avoided [38]. National guidance on the risk conferred by a HPV result in women with low-grade screening may reduce the dominant role of affect and strengthen the cognitive component of decision-making. Moreover, studies which assess the benefit of HPV genotyping in this cohort may also assist in risk stratification as the 10-year incidence of CIN3 is 17% with HPV 16, 14% with HPV 18 and 3% with other high-risk subtypes [39].

The study group identified discrepancies in the recommended technique and setting of cytological follow-up. Colposcopy nurses preferred community follow-up and this may be a reflection of the higher volume of patients they see. Whereas the majority of Doctors favoured colposcopy follow-up and this attitude may be influenced by the higher proportion of women with cervical cancer they manage. Although current evidence suggests an increased cytological yield when using a cytobrush in combination with a Cervex-Brush (which samples cells from inside and outside of the cervix) [40], there is a paucity of evidence correlating this increased yield of cells when used in conjunction with unsatisfactory colposcopy [41]. This lack of knowledge and the inability of community services to offer a cytobrush compounded decision-making, particularly for doctors. Studies which improve knowledge in this area may aid rational judgement.

Patient choice was cited as a major influence affecting decision-making. Eighty one percent of referrals to colposcopy are for low-grade screening results [3] but patients report the same level of anxiety irrespective of the cytological grade [14]. This anxiety is driven by fear of cancer, worries that subsequent cytology will be abnormal and future fertility concerns [14, 42]. There is a plethora of literature assessing women’s preferences for the management of low-grade cytology when colposcopy is satisfactory, with the majority of studies showing a preference for colposcopic review over cytological (cervical smear) surveillance [43, 44]. Furthermore, if cytological follow-up is chosen, women have cited a preference for ‘regular’ screening [45]. Until such time as one outcome is shown to be superior to another it could be argued that Colposcopists should advocate the more cost-effective approach of cytological follow-up. However, in a shared care model, determining patient preferences will improve patient satisfaction and outcomes [46] – even if this involves, as shown in our study, young women with low-grade screening and low risk factors choosing excisional treatments over cytological follow-up.

To reduce the emotional burden of decision-making, health care providers will defer decision-making [47] and the majority of our participants felt a reduction in emotional burden following an MDT decision to offer excision, particularly in young and/or nulliparous women. This finding is supported by studies which have shown that the use of the MDT reduces overtreatment in the colposcopy setting [48] and potential heterogeneity in care. Although it should be noted, that with a paucity of evidence to guide this expert body’s management, homogeneity of care may be achieved within departments or regions but may not occur at a national level.

In areas that lack evidence it is clear that prior expertise forms the basis of decision-making [49]. This was evident when participants recommended a depth of excision which was incongruent with recent national guidance [7]. Furthermore, experienced Colposcopists were more likely to recommend longer total length of conservative follow-up and at 12 month intervals in women with low-grade screening. Evidence has suggested that experts are ‘wise risk takers’ [50], their knowledge reduces anxiety and indecision allowing them to make decisions which deviate from set guidance to individualise care [51].

Colposcopists are independent practitioners and it could be argued that guidance may not be necessary in scenarios which lack consensus of opinion. Furthermore, it is clear that not all clinical scenarios can conform to guidance and removing all uncertainty from the medical profession may hinder adaptability, critical analysis, maturity of thought and patient choice. That being said, part of a clinicians’ duty is to reduce patient anxiety and optimise clinical outcomes, but how can this be achieved if the clinician themselves is plagued by anxiety. In situations where there is a lack of clear evidence, affect may compromise rational judgements. Homogeneity of care improves service provision and clinical outcomes through consistent use of evidenced based interventions [52] and the majority of decisions in colposcopy contain a small number of variables and are fairly unambiguous. Guidelines improve decision-making in areas of ambiguity, recognize shortfalls in the literature, provide assurance that clinicians are advocating appropriate treatments and promote under-recognised and neglected patient cohorts.

This study had some limitations. Assessing practice in one geographical UK region may increase the institutional bias but the inclusion of four centres with varying patient populations and participants who trained in different regions improved the generalizability of the data. Moreover, there was no difference in opinion based on training location. To triangulate these findings we propose a nationwide survey, based on the themes identified, to explore the frequency of these opinions and identify areas of consensus where guidance may be clarified. It is also important to consider why participants agreed to take part; it could be argued that attendees did so to express a particular viewpoint and therefore the data may not resonate with national opinions. However, only four of twenty eight Colposcopists did not participate and this was due to conflicting clinical commitments. Furthermore, two of these gave written statements for clinical scenarios that they wished to be discussed. Although these statements were not used in the analysis, they stimulated animated discussions on the optimal cytology collection device and the risks and benefits of repeating the referral cytology at the first Colposcopy appointment.

Age and gender were not collected for confidentiality reasons. However, gender has been shown to influence clinical decision-making. Female clinicians can have longer consultation times with more time devoted to counselling [53] whereas male colleagues may spend more time discussing technical aspects [54]. As a key outcome of this study was factors affecting choice of conservative or surgical management, assessing this association would be useful for guideline implementation and should be explored in future studies such as a national survey. Correlation of experience with management decisions may also be valuable for guideline development and it could be argued that more weight should be applied to recommendations from Colposcopists who have more experience in this area. However, measuring competence and experience can be problematic; most decision-making in Colposcopy contains limited variables and is formulaic, all Colposcopists have to attain the same basic competencies and re-accredit three yearly. Therefore a consensus opinion may provide best recommendations for guidance and in areas of dissonance, further research will be required to aid analytical thinking and reduce uncertainty.

Focus groups, rather than interviews or questionnaires, were chosen as the method of study as numerous viewpoints on a specific issue can be studied in an interactive setting and comments made by individual participants stimulated group discussions. The use of focus groups reduced the interaction of the facilitator and the input or potential bias of the researchers. Moreover, they provided richer data than a questionnaire by expanding upon the decision-making process and enabling targeted suggestions for guidance which was the key component of interest in this study.

Block sampling was chosen as it can be more representative; a heterogenous group ensured differing opinions were shared leading to lively debates in some of the units. Sensitization, with the possibility of pre-set answers which may reduce analytical thinking during the focus groups, was reduced by the provision of a general theme in the participant information sheet rather than set questions. Further strengths included the use of open ended questions, an extensive coding process and an iterative analysis which helped ensure saturation and depth of information was attained. Transcription of the data by KM facilitated reading and interpretation of the data as suggested by Braun and Clarke. None of the participants withdrew their data and the respondents verified the validity of the transcripts. The interpretivist method of data collection, the heterogenous group, double coding of the transcripts and a self-awareness of the researchers own preconceptions by including both Colposcopists and qualitative researchers in the study group will have reduced reflexitivity [55].

## Conclusions

We would argue that in order for experts to analyse information they utilise cognition as well as the emotional impact from their past experiences. However, in areas of clinical uncertainty when decisions are dominated by emotive factors, clinical guidance can reduce the difficulty and anxiety of decision-making. Colposcopists’ opinions and experiences are likely to have a significant effect on national policy and the implementation of guidelines in a clinical setting. In our study, clear areas of consensus included use of the multidisciplinary team and offering excision to women with high-grade cytology and high risk women with low-grade cytology such as smokers, non-attenders, parous and older women. Areas of dissonance, which were affected by paucity of evidence and anxiety of missing a cancer, deterred long-term conservative follow-up and promoted repetition of the referral cytology and higher than desired treatment rates in women with low-grade screening.

Future research should focus on a nationwide survey to explore the frequency of opinions identified in this study, assessment of the optimal cytological collection device for a transformation zone type 3, including efficacy of primary care sampling in this cohort. Prospective studies which assess the progression rate of pre-cancerous change in women with low-grade cytology to inform interval lengths in women who are managed with cytological follow-up, evaluation of patient decision-making and techniques that improve the PPV of screening in this cohort such as biomarkers that detect HPV-transforming infection, HPV genotyping and techniques which sample an endocervical transformation zone.

## Abbreviations

HPV: Human papillomavirus
LLETZ: Large loop excision of the transformation zone
MDT: Multidisciplinary team
TA: Thematic analysis
TZ type 3: Transformation zone type 3; cells that are infected by HPV but not visible for assessment as they are tucked inside the cervical canal
